# Continuous Skin Rejuvenation by Combining Nonablative Fractional Laser With Daily Application of a Multibeneficial Composition Formulation: A Blinded Randomized Clinical Trial Study

**DOI:** 10.1002/hsr2.70423

**Published:** 2025-03-02

**Authors:** Xinxuan Zhang, Manru Ning, Mengqing Lin, Qi Tang, Yihuai Liang, Feifei Wang, Xiaoke Xu

**Affiliations:** ^1^ Xiaoke BeauCare Clinic Shenzhen Guangdong China; ^2^ Yunnan Characteristic Plant Extraction Laboratory Co. Ltd. Kunming Yunnan China; ^3^ Yunnan Botanee Bio‐Technology Group Co. Ltd. Kunming Yunnan China

**Keywords:** anti‐aging, formulation, non‐ablative fractional laser, skin barrier, skin physiology, skin wrinkles

## Abstract

**Background and Aims:**

Skin aging is a common concern among individuals, and laser treatments are recognized as one of the most effective approaches to mitigate the aging process. The study aims to compare a multibeneficial formula serum versus a blank formulation in achieving maximum efficacy following a single treatment of nonablative fractional laser for facial skin rejuvenation.

**Methods:**

This study was a double‐blind, split‐face, monocentric, randomized clinical trial in China (September 24, 2023–March 07, 2024), and 37 patients seeking the Fotona 4D laser treatment for aging‐related facial changes were enrolled. After one full‐face laser treatment, each patient applied the test serum to one side and the blank formulation to the other, randomly, twice daily for 28 days. Two dermatologists assessed facial skin quality and aging signs at baseline and Day 0 (D0, immediately after the laser treatment), D3, D7, D14, and D28. Noninvasive measurement and self‐assessment questionnaires were also administered at each visit. According to the types of variables, appropriate statistical tests, including the Friedman test, ANOVA test, and Wilcoxon signed‐rank test, were used to examine the within‐groups or between‐groups differences.

**Results:**

Thirty‐three women, aged 35–49 years, completed the study. After 28 days of the test serum application, the visual clinical scores rated by investigators showed more significantly beneficial changes on the test side than those on the control. More significant improvements in index parameters for the test sides were also found both in wrinkles with a 21.14% decrease of SEw value from the baseline and in elasticity with a 14.99% decrease of R2 value, while the corresponding reductions were 3.83% for SEw and 4.10% for R2 found on the control sides. The reduction of the nasolabial folds area proportion, analyzed by Primos, was 10.61% on the test sides and 3.39% on the control. No adverse events were reported.

**Conclusion:**

The serum with a multi‐beneficial composition can contribute to achieving a more significant and sustainable efficacy after the Fotona 4D treatment in skin rejuvenation improvement.

ClinicalTrials.gov ID: NCT06140628.

## 1 Introduction

The skin, as the body's largest organ, provides a physical barrier against environmental factors and is exposed to damage throughout life [1]. Skin aging is influenced by both intrinsic and extrinsic factors [2]. Intrinsic aging is primarily determined by genetics and age, while extrinsic aging is characterized by fine lines, pigmentation, and roughness [3]. Since facial skin visibly shows signs of aging, it is of significant concern to many people. Although intrinsic aging is irreversible, photoaging can be mitigated through various protective measures. Therapies for photodamage [4], such as non‐ablative fractional laser (NAFL), intense pulsed light (IPL), and radio‐frequency light (RFL), have become increasingly popular. Additionally, incorporating skincare products into daily routines, including cleaning, moisturizing, and sun protection, can effectively protect the skin and slow the aging process.

Fotona 4D, an NAFL with two complementary wavelengths (2940 nm and 1064 nm), treats four distinct dimensions of the skin to rejuvenate it from the inside out. It is particularly effective for tightening lines around the eyes, though temporary pain, redness, and swelling may occur, affecting postoperative comfort.

Because of the desire for improved appearance with shorter recovery times and longer‐lasting effects, the concept of “integrated skincare” has emerged [5]. This approach combines clinically proven skincare products with professional medical esthetics to enhance treatment benefits. Studies have shown that “integrated skincare” can synergistically reduce adverse reactions and promote skin barrier repair [6]. Numerous botanical extracts with antiaging properties have been identified, targeting various aspects of aging. For example, *Cordyceps sinensis* extract [7], *Paeonia suffruticose* extract [8], and ergothioneine [9] regulate the extracellular matrix; *Kappaphycus alvarezii* extract [10] limits the telomere shortening and slows the senescence of dermal fibroblasts; *Acmella oleracea* extract [11] relaxes muscle to recuperation of contractile activity; and *Morus alba* extract inhibits mitogen‐activated protein kinases (MAPKs). Furthermore, a recent preliminary study demonstrated that daily use of a multi‐beneficial composition formulation can promote facial rejuvenation [12]. Based on the efficacy of Fotona 4D for facial rejuvenation and the concept of “integrated skincare,” this study aimed to systematically evaluate the short‐ and long‐term effects of Fotona 4D treatment alone and in combination with a multi‐component antiaging composition formulation. The assessment was conducted using clinical evaluations, noninvasive methods, and self‐assessment questionnaires. The short‐term effect on the epidermal barrier was measured by transepidermal water loss (TEWL), while the long‐term effects on facial skin rejuvenation were evaluated across five parameters including wrinkles, firmness, smoothness, roughness, and elasticity.

## 2 Methods

### 2.1 Ethics

The study was approved by Shanghai Ethics Committee for Clinical Research (Approval Number: SECCR/2023‐101‐01). Before any study‐related procedures or measurements, written informed consent was obtained from all participants. Participants were informed about the purpose of the study, procedures involved, potential risks, and their right to withdraw at any time without any consequences.

### 2.2 Study Design

This study was a double‐blind, split‐face, monocentric, randomized clinical trial. Thirty‐seven participants were enrolled and underwent a 2‐week washout period using basic skincare products, and 33 of them received the Fotona 4D laser treatment and completed the study. The skin biophysical parameters, objective assessments, and self‐assessments were evaluated on D0, D3, D7, D14, and D28 use after the Fotona 4D laser treatment.

### 2.3 Participants

Participants meeting all inclusion criteria and none of the exclusion criteria were enrolled and provided informed consent. Inclusion criteria: (1) Healthy female aged 30–50; (2) subjects with under‐eye wrinkles, pore appearance, and forehead wrinkles (scores are all > 2 from the *Asian Skin Aging Atlas, Volume 2* [13]; (3) subjects with nonsensitive skin; (4) subjects are willing to use the test serum on split‐face for 28 days; (5) subjects with no history of antibiotics in the past 3 months; and (6) subjects understand the nature of the study and sign the informed consent form (ICF). Exclusion criteria: (1) Subjects with known intolerance or hypersensitivity to skincare ingredients; (2) female subjects who are pregnant, lactating or plan to become pregnant; (3) subjects who have received esthetic medical treatment (photoelectric, tightening, filling, etc.) within the past 6 months; (4) subjects with erythema, rash, flaking and edema on faces; (5) subjects who participated in clinical trials within 1 month; (6) subjects with antiallergy medication history within 1 month; and (7) any subjects those the investigator considers ineligible.

### 2.4 Laser System

Each participant received a single laser treatment (Fotona 4D Pro, Fotona d.o.o., Slovenija) on D0 using the following three modes:

FRAC3 Mode: 4 mm spot size, 20–25 J/cm² fluence, 1.6 s pulse width, a frequency of 4.0 Hz, and 1500 rounds at each session.

PIANO Mode: 9 mm spot size, 120–150 J/cm² fluence, 5 s pulse width, a frequency of 4.0 Hz, and 1500 rounds at each session.

Superficial Mode: 5 mm spot size of 5 mm, 1.0–1.5 J/cm² fluence, and a frequency of 2.0 Hz.

### 2.5 Treatment Protocol

All enrolled participants were instructed to discontinue their usual skincare products for 2 weeks before the treatment. Basic skincare products, that is, sunscreen, cleansing foam, and moisturizing emulsion, were provided for daily use throughout the study.

On D0, all participants' facial skin was cleansed with a neutral lotion before the session, and no topical anesthesia was applied. Each of them lying in a horizontal position, received the three modes of laser therapy, successively. Immediately after the laser treatment, they applied the antiaging formulation on the test side and the blank formulation on the control side, maintaining this regimen twice daily for 28 days. The facial midline, defined by anatomical landmarks such as the glabella, nasal bridge, philtrum, and chin, served as the physical boundary between the two sides.

### 2.6 Formulation

The test item is a commercially available antiaging serum, produced by Yunnan Botanee Co. Ltd in China, containing a proprietary complex of *C. sinensis* extract, *P. suffruticosa* extract, *K. alvarezii* extract, ergothioneine, *A. oleracea* extract, *M. alba* extract, β‐alanyl hydroxyprolyldiaminobutyroyl benzylamide, and palmitoyl hexapeptide‐12. The placebo control is a base formulation without the mentioned complex.

### 2.7 Instrumental Evaluation

All outcomes were assessed under controlled environmental conditions (21°C ± 1°C with 50% ± 10% relative humidity). Participants acclimated for 30 min before any evaluation.

Skin surface microstructure and wrinkles were investigated using PRIMOS Clinical Research System (Canfield Scientific), measuring depth and area percentage of crow's feet wrinkles and nasolabial folds at each visit. Skin texture was recorded using VisioScan VC20 plus (Courage & Khazaka), analyzing parameters such as skin smoothness (SEsm) and wrinkles (SEw). Skin elasticity was determined by the Cutometer MPA 580 (Courage & Khazaka), focusing on R2 (total elasticity) and F4 (firmness). Trans‐epidermal water loss (TEWL) was measured using Tewameter (Courage & Khazaka). The VISIA system (Canfield Scientific) was used for capturing facial images, and skin thickness was determined by Ultrascan UC22 (Courage & Khazaka).

### 2.8 Clinical Evaluation

Clinical evaluations were conducted during all visits by two independent dermatologists using the *Evaluation Criteria for Human Skin Aging* and *Asian Skin Aging Atlas, Volume 2*. The *Evaluation Criteria for Human Skin Aging* scores forehead wrinkles from 0 to 8, crow's feet wrinkles from 0 to 6, under‐eye wrinkles from 0 to 9, and nasolabial folds from 0 to 7. The *Asian Skin Aging Atlas, Volume 2* scores cheek sebaceous pores from 0 to 5.

Clinical evaluations also included a 10‐point visual analog scale (VAS, 0–9) for attributes such as elasticity, roughness, firmness, and global face wrinkles, conducted by the two dermatologists.

The Global Esthetic Improvement Scale (GAIS) was used at each visit after the Fotona 4D treatment to assess facial improvement on a 5‐point scale: 3 = improved completely; 2 = improved significantly; 1 = improved slightly; 0 = no difference; −1 = worsened. All participants were asked to complete a satisfaction questionnaire regarding the antiaging effects of the treatment for both face sides, with answers recorded on a 9‐point scale (1 = completely disagree, 9 = totally agree).

### 2.9 Statistical Analysis

Statistical analysis was performed by using SPSS version 22.0. The categorical variables were reported as counts and percentages, while the continuous variables were reported in the form of mean ± standard deviation (SD) or as the minimum, maximum, and median. The Shapiro–Wilk test was used to assess the normality of the data set. The intra‐group comparison was performed between the baselines and the other visits, using the analysis of variance (ANOVA) test for data normally distributed, otherwise, the Friedman test would be used. Intergroup comparisons were performed between the treatment and control sides. Independent samples *t*‐test was used for data with normal distribution, and the Mann–Whitney *U* test was used for the non‐normally distributed data. Additionally, the Mann–Whitney *U* test and the Friedman test were applied to the categorical variables for comparing the between‐group differences and the within‐group ones, respectively. A two‐sided *p*‐value less than 0.05 was considered to indicate statistically significant.

## 3 Result

### 3.1 Demographic Characteristics of Subjects

A total of 37 females were included and 33 of them finished the study, with two withdrawing during the washout period and another two withdrawing after the Fotona 4D treatment. The mean age of the 33 participants was (43.45 ± 3.94) years (range: 35–49 years).

### 3.2 Clinical Evaluation of Overall Improvement (GAIS)

**Figure 1:**
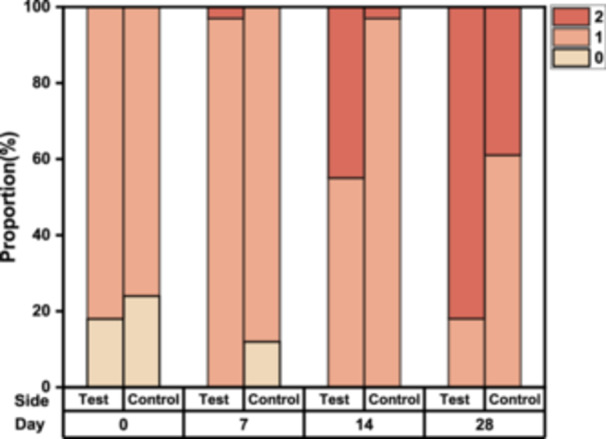
Global Esthetic Improvement Scale (GAIS) scores for skin aging on the test and control sides at baseline, on D7, D14, and D28.

During the study period, the GAIS scores gradually increased on both facial sides. The distribution of GAIS scores indicated that overall improvement in facial skin was more prominent on the test side than the control side, with statistical significance observed from D4 to D28 (Figure 1). On the test side, all participants achieved at least an “Improved” rating by D7, whereas the same effect was observed starting from D14 on the control side. At the final follow‐up visit on D28, 27 cases (82%) had a GAIS score of 2, and six cases (18%) scored 1 on the test side; correspondingly, 13 cases (39%) scored 2, and 20 cases (61%) scored 1 on the control side.

### 3.3 Improvement in Skin Barrier

**Figure 2:**
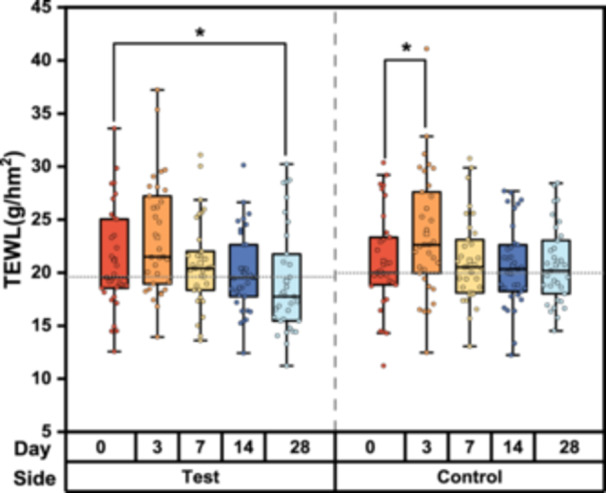
Changes in transepidermal water loss (TEWL) over time among participants using the test or control items. Data were analyzed using the Friedman test; *p < 0.05, compared with the baseline.

The measurement of TEWL on the skin surface provides insights into the barrier function of the stratum corneum. Figure 2 illustrates the TEWL on the cheek before and after 28 days of applying the test or control items. TEWL values on the control side were significantly higher than the baseline on D3 (*p* < 0.02), indicating an imperfect skin barrier, and showed only a 1.58% decrease from the baseline on D28. However, on the test side, the TEWL values gradually decreased to baseline levels by D7 and further decreased by 9.51% at the end of the study (*p* < 0.001), which suggested the test item may help accelerate skin barrier recovery and enhance skin barrier integrity.

### 3.4 Improvement in Antiwrinkle

#### 3.4.1 Clinical Evaluation Based on a 10‐Point VAS (0–9)

**Figure 3:**
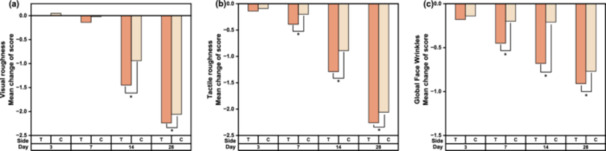
Bilateral comparison of the clinical evaluation scores at each visit relative to baseline. (a) Mean change in visual roughness. (b) Mean change in tactile roughness. (c) Mean change in global face wrinkles. Data were analyzed using the Wilcoxon Rank Sum Test test; *p < 0.05, compared with both sides.

The VAS evaluation, where a higher score indicates a worse condition, revealed a decreasing trend in scores on both sides with each successive visit. A bilateral comparison of the VAS scores for roughness and global face wrinkles at each visit relative to the baselines is shown in Figure 3. Statistically significant differences in score changes between the test and control sides were observed in tactile roughness and global wrinkles starting from D7, while in visual roughness from D14.

#### 3.4.2 Clinical Evaluation Based on Atlas

**Figure 4:**
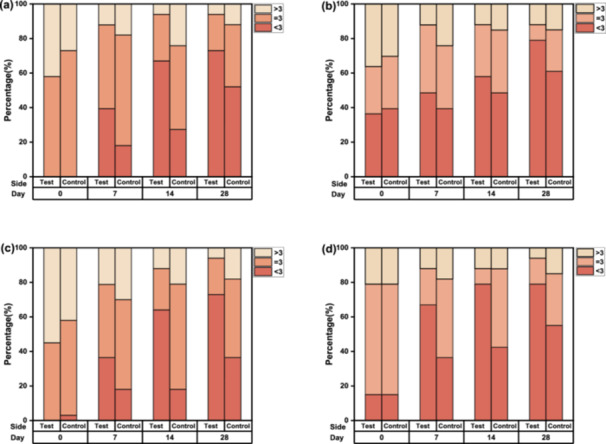
Clinical evaluation based on atlas scores on D7, D14, and D28 showing the percentage of scores on each side. (a) Forehead wrinkles. (b) Nasolabial fold wrinkles. (c) Underneath eye wrinkles. (d) Crow's feet wrinkles.

The distribution of scores across different parameters was similar, with a median of about 3 points (Figure 4). Therefore, the analysis compared the improvement on both sides using a cutoff of 3 points. The proportion of scores related to wrinkle parameters indicated a trend of improvement on both sides from D7 to D28.

In comparison to their baselines, statistically significant changes in each clinical efficacy indicator were demonstrated on both sides by D28, except for the nasolabial folds of the control side. Daily use of the test serum resulted in improvements in under‐eye wrinkles, crow's feet, forehead wrinkles, and nasolabial folds as early as D7. On the control side, significant improvement in under‐eye wrinkles was observed by D14, while crow's feet and forehead wrinkles showed significant improvement only by D28.

#### 3.4.3 Measurement of Skin Texture

**Figure 5:**
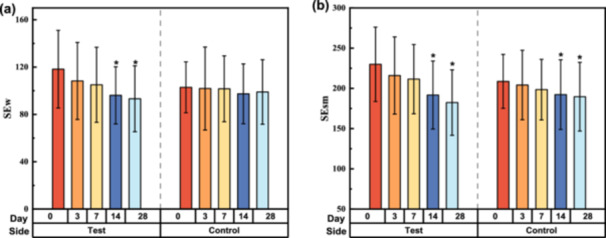
Evolution of skin wrinkle parameters by skin biophysics analysis after 28 days of using test or control products. (a) Mean changes in SEw (mean ± SD). (b) Mean changes in SEsm (mean ± SD). Data were analyzed using the Friedman test; *p < 0.05, compared with the baseline. SD, standard deviation.

The treatment with the test serum contributed to a significant reduction in the facial wrinkle parameter (SEw). From the baseline, the test side achieved a reduction by 21.14% over 28 days, whereas the control side showed only a slight antiwrinkle effect, with a decrease by 3.83%. The mean SEw improvement of the test side was more significant than that of the control side on both D14 and D28 (*p* < 0.002) (Figure 5). Especially, the mean SEsm of the test side decreased by 20.68% on D28, that is, from 229.98 at baseline to 182.41, while the control side showed a decline from 208.73 to 189.68, a reduction by 9.13% (Figure 5).

#### 3.4.4 Measurement of Skin Wrinkles

**Figure 6:**
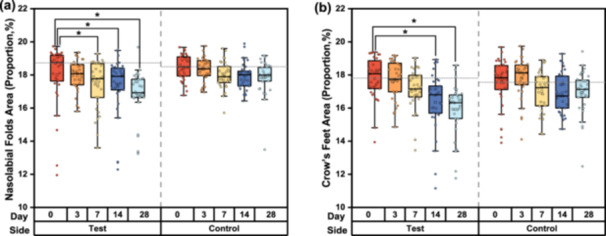
Evolution of skin wrinkle parameters by skin biophysics analysis after 28 days of using test or control products. (a) Changes in the distribution of data in the nasolabial folds wrinkles area. (b) Changes in the distribution of data in the crow's feet wrinkles area. Data were analyzed using the Friedman test; *p < 0.05, compared with the baseline.

The area proportion of the nasolabial folds on the test side decreased significantly by D3 and this decline trend was sustained through D28, with a decrease by 7.03% from 18.00% at baseline to 16.73% on D28. However, a slight decrease by 3.36% occurred to the control side, from 18.47% to 17.85% (Table 1 and Figure 6). Additionally, a similar pattern was found for changes over time in the area proportion of crow's feet (Table 1 and Figure 6).

**Table 1:** Changes associated with skin wrinkles and skin thickness parameters during the test.

		Side	Baseline	D3 (△1)	D7 (△2)	D14 (△3)	D28 (△4)
Percentage of wrinkle area (%)	Nasolabial folds	Test	18.00% ± 0.33%	17.95% ± 0.16% (−0.27%)	17.39% ± 0.25% (−3.36%)	17.32% ± 0.31% (−3.79%)	16.73% ± 0.27% (−7.03%)
		Control	18.47% ± 0.13%	18.34% ± 0.12% (−0.70%)	17.95% ± 0.14% (−2.81%)	17.94% ± 0.14% (−2.88%)	17.85% ± 0.18% (−3.36%)
	Crow's feet	Test	17.42% ± 0.38%	17.33% ± 0.39% (−0.47%)	16.69% ± 0.41% (−4.15%)	15.98% ± 0.42% (−8.24%)	15.43% ± 0.49% (−11.41%)
		Control	17.56% ± 0.26%	17.85% ± 0.22% (+1.68%)	17.06% ± 0.22% (−2.81%)	16.94% ± 0.21% (−3.54%)	16.98% ± 0.24% (−3.28%)
Wrinkle depth (μm)	Nasolabial folds	Test	77.36 ± 4.05	74.36 ± 2.90 (−3.88%)	74.24 ± 3.26 (−4.03%)	73.30 ± 2.89 (−5.25%)	72.39 ± 2.64 (−6.42%)
		Control	76.52 ± 4.78	76.12 ± 4.11 (−0.51%)	75.03 ± 3.26 (−1.94%)	73.64 ± 3.43 (−3.76%)	73.21 ± 3.01 (−4.32%)
	Crow's feet	Test	56.18 ± 3.72	53.15 ± 3.37 (−5.39%)	52.48 ± 3.05 (−6.58%)	51.58 ± 3.01 (−8.20%)	51.48 ± 2.85 (−8.36%)
		Control	59.88 ± 6.11	56.58 ± 5.07 (−5.52%)	57.09 ± 5.14 (−4.66%)	56.76 ± 5.68 (−5.21%)	57.30 ± 5.91 (−4.30%)
Dermal thickness		Test	1794.42 ± 40.78	1748.82 ± 48.93 (−2.54%)	1885.97 ± 42.96 (+5.10%)	1925.85 ± 49.62 (+7.32%)	2009.45 ± 46.26 (+11.98%)
		Control	1725.21 ± 44.75	1680.76 ± 50.30 (−2.58%)	1845.15 ± 46.02 (+6.95%)	1879.67 ± 42.66 (+8.95%)	1938.79 ± 49.16 (+12.38)

*Note:* Data at baseline, Day (D)3, D7, D14, and D28 were indicated as mean ± standard deviation (SD); Δ1, the percentage changes on D3 (vs. baseline); Δ2, the percentage changes on D7 (vs. baseline); Δ3, the percentage changes on D14 (vs. baseline); Δ4, the percentage changes on D28 (vs. baseline). Data related to wrinkles were analyzed using the Friedman test, and data related to dermal thickness were analyzed using the analysis of variance (ANOVA) test.

**Figure 7:**
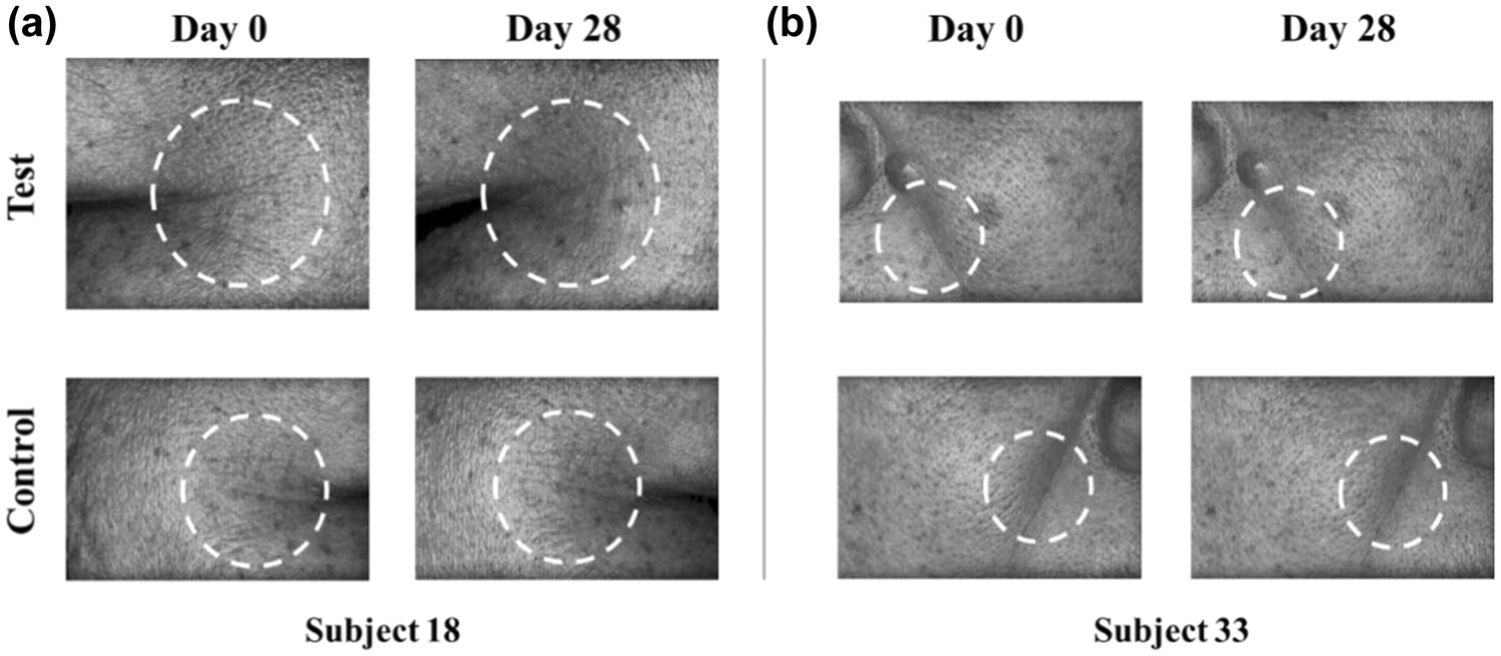
Representative three‐dimensional images of wrinkle changes at baseline and on D28. (a) Three‐dimensional images for crow's feet wrinkles on the test and control sides in Subject 18. (b) Three‐dimensional images for nasolabial folds wrinkles on the test and control sides in Subject 33.

Wrinkle depth is another indicator of evaluating the antiwrinkle effect. Both crow's feet and nasolabial folds showed a decreasing mean depth over 28 days, although not statistically significant compared to the baseline (Table 1). As illustrated in Figure 7, representative examples of 3D images exhibited wrinkle changes within the dotted circles.

### 3.5 Improvement in Skin Firmness

#### 3.5.1 Clinical Evaluation Based on a 10‐point VAS (0–9)

**Figure 8:**
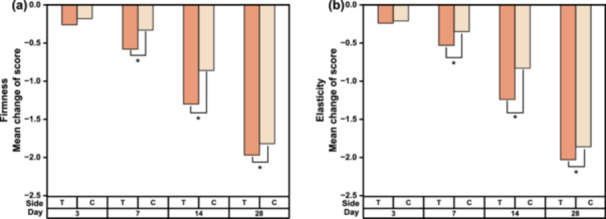
Bilateral comparison of clinical evaluation scores at each visit relative to the baseline. (a) Mean change in firmness. (b) Mean change in elasticity. Data were analyzed using the Friedman test; *p < 0.05, compared with both sides.

The scores of firmness and elasticity graded visually by dermatologists were falling with each successive visit for both sides (Figure 8). A bilateral comparison of the scores relative to the baseline showed statistically significant lower scores were observed on the test side than the control side early as on D7.

#### 3.5.2 Measurement of Skin Mechanical Properties

**Figure 9:**
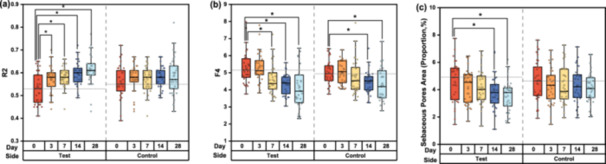
Evolution of skin firmness parameters by skin biophysics analysis after 28 days of using test or control products. (a) Changes in R2. (b) Changes in F4. (c) Changes in the proportion of sebaceous pores area. Data for R2 and sebaceous pore area distribution were analyzed using analysis of variance (ANOVA), while F4 data and the percentage of sebaceous pores area were analyzed using the Friedman test; *p < 0.05, compared with the baseline.

The increasing values in R2 and decreasing ones in F4 from baselines were demonstrated on both sides (Figure 9). Notably, the test sides suggested a more pronounced improvement in the two measured indices than their counterparts.

#### 3.5.3 Measurement of Dermal Thickness

**Figure 10:**
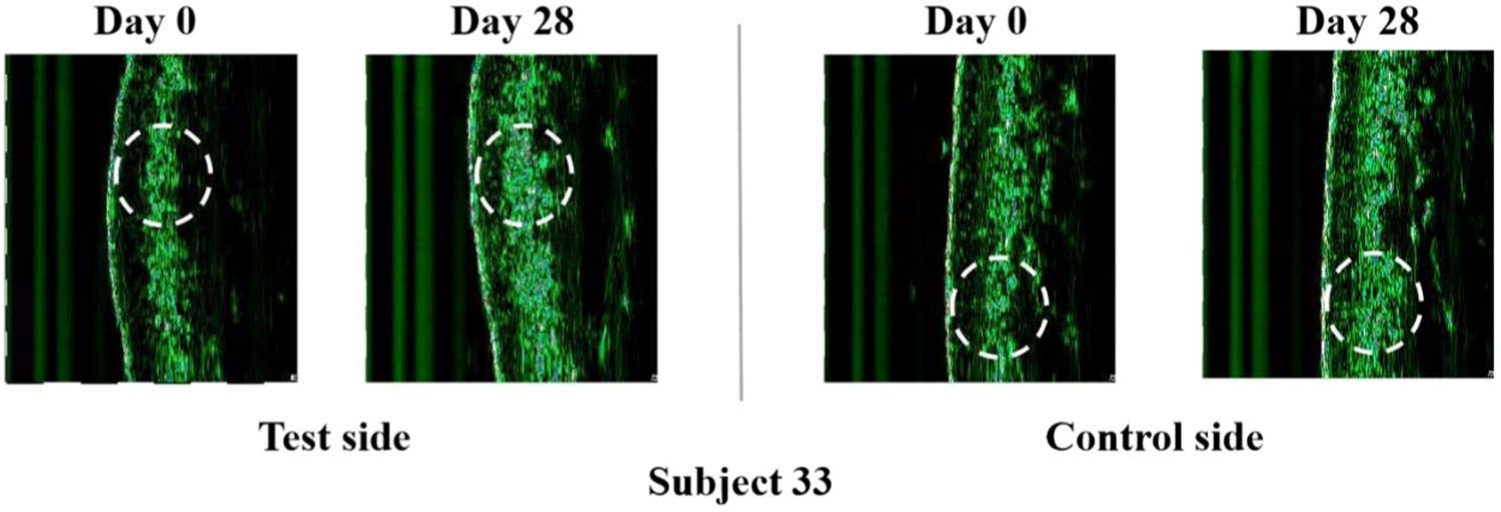
Images of skin condition at baseline and on D28. Ultrasonographic skin aspect on the test and control sides in Subject 33, the white circle represents the dermis.

The dermis of the cheeks, indicated by the ultrasonographic measurement, becomes thicker on D14 and D28 for both sides (Table 1). There was a greater average magnitude of thickness elevated from baselines on the test sides compared with the control ones. The representative examples of ultrasonographic skin images are shown in Figure 10.

#### 3.5.4 Evaluation of Sebaceous Pores

**Figure 11:**
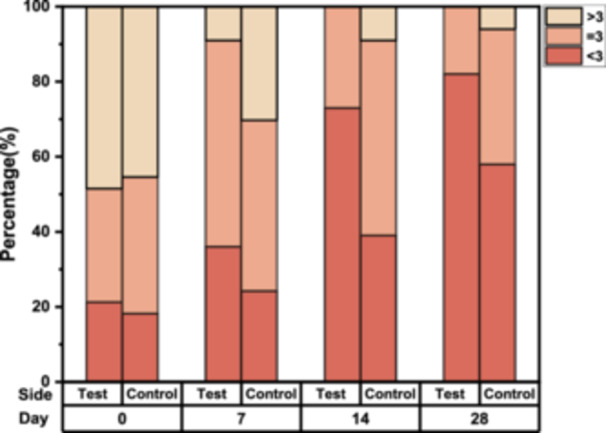
Clinical evaluation based on atlas related to sebaceous pores at D7, D14, and D28 showing the percentage of score at each side.

The changes in sebaceous pores, as reductions in pore size and pore area percentage reflect firmer skin, can be an alternative indicator of skin firmness. Low sebaceous‐pores scores (≤ 3) based on the atlas rose in proportion for both sides throughout the study (Figure 11). Additionally, larger proportions of low scores appeared on the test side than on the control one from D7 on.

**Figure 12:**
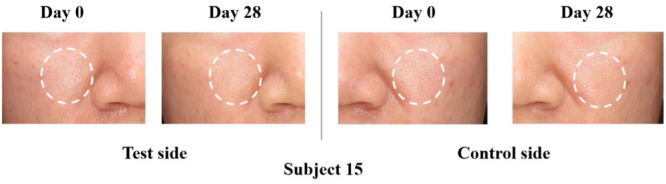
Images of skin condition at baseline and on D28. Standard optical image of sebaceous pores on the test and control sides in Subject 15.

The area percentages of sebaceous pores produced by PRIMOS were depicted in Figure 9. After 28 days, the test side exhibited a reduction in mean area percentage by 22.49%, while the control one did by 11.65% only. Signs of improvement were observed on the test sides at visits of D14 (*p* < 0.02) and D28 (*p* < 0.001). The representative example images of the sebaceous pores are shown in Figure 12.

### 3.6 Self‐Assessment

Each participant completed a questionnaire on D7, D14, and D28, and revealed self‐perceived benefits brought by the test serum consistent with expert visual grading and noninvasive biophysics measurement.

### 3.7 Adverse Events

No serious adverse events were observed during the entire study. None of the participants suffered erythema or papules due to the laser treatment and daily use of test serum. Only one case of very mild edema was reported, and the symptom was considered secondary to the laser treatment and relieved spontaneously within 3 days.

## 4 Discussion

Participants enrolled in this study sought the Fotona 4D laser treatment due to concerns about aging‐related appearance changes. Therefore, optimizing the efficacy of skin rejuvenation and minimizing adverse effects from cosmetic laser therapy is crucial.

Compared with traditional fractional lasers, NAFLs can enhance skin quality with fewer adverse effects and reduced downtime. Nevertheless, the three modes employed in this study may still induce mild erythema, edema, flushing, pimples, and desquamation. The active ingredients, including *C. sinensis* extract [7], *M. alba* extract [14], and ergothioneine [15], incorporated into the test serum, can counteract oxidative stress and stimulate collagen renewal. This efficacy was confirmed by the TEWL values and tolerability assessments.

Furthermore, penetration of the epidermal barrier has always been a significant challenge for active ingredients in drugs and cosmetics, with the stratum corneum being the primary rate‐limiting step [16, 17]. Multiple studies showed that laser pretreatment can enhance the permeability and depth of molecule penetration [18, 19, 20]. The superficial mode used in this study is a type of micro‐ablative fractional laser that may facilitate deeper penetration, allowing active ingredients to reach deeper skin layers through ablated channels extending into the dermis.

Previous research has shown that dermal density and the thickness of the epidermis and dermis are key parameters in demonstrating the cutaneous regeneration process [21]. The test serum significantly improved most measured aging parameters, with these improvements sustained over 28 days and progressively enhanced from baseline on the test sides compared to the control sides. The gradual increase in dermal density and thickness is probably facilitated by the addition of *Cortex moutan* extract [8], which induces fibroblast synthesis and sustains the effects of the Fotona 4D laser, thereby prolonging the benefits of facial rejuvenation achieved through medical esthetics.

However, the study's limitations include a small sample size and a relatively short follow‐up period. A larger sample size could enhance the reliability of the study and strengthen the statistical power of the analysis. Extending the follow‐up period is also helpful in exploring the sustainable benefits of product use on skin rejuvenation. In addition, common adverse events associated with lasers, such as erythema, desquamation, and burning, were infrequent in this study. Some anti‐inflammatory ingredients like *Portulaca oleracer* extract, *Helichrysum stoechas* [22] extract, and *M. alba* extract [23, 24, 25] are included in the formulation, but whether the test serum can reduce inflammation is still unclear and needs to be proved in the future research.

Overall, the serum with a multi‐component antiaging composition enhances the efficacy of Fotona 4D in skin rejuvenation. However, more solid evidence originating from well‐designed clinical trials is necessary.

## Author Contributions

**Xinxuan Zhang:** conceptualization, investigation, writing – review and editing. **Manru Ning:** conceptualization, supervision, writing – original draft. **Mengqing Lin:** investigation. **Qi Tang:** investigation. **Yihuai Liang:** conceptualization, methodology, writing – review and editing. **Feifei Wang:** funding acquisition, writing – review and editing, project administration. **Xiaoke Xu:** conceptualization, investigation, methodology, project administration, writing – review and editing.

## Conflicts of Interest

The authors declare no conflicts of interest.

## Transparency Statement

The lead author Feifei Wang, Xiaoke Xu affirms that this manuscript is an honest, accurate, and transparent account of the study being reported; that no important aspects of the study have been omitted; and that any discrepancies from the study as planned (and, if relevant, registered) have been explained.

## Data Availability

The data that support the findings of this study are available on request from the corresponding author. Study Protocol and all of the individual participant data collected during the trial, after deidentification can be shared, immediately following publication.
